# Identification of a TBHQ-Interfering Peak in Crude Canola Oil Using AOCS Official Method Ce 6-86 and its Chromatographic Resolution

**DOI:** 10.1007/s11746-017-3039-2

**Published:** 2017-08-30

**Authors:** Michael R. Blumhorst, Travis Mahan, Kathryn Stanley, Aaron Griffith, Mark W. Collison

**Affiliations:** Research Division, Archer Daniels Midland Company, James R. Randall Research Center, 1001 N. Brush College Road, Decatur, IL 62521 USA

**Keywords:** Canolol, TBHQ, Canola, Rapeseed

## Abstract

AOCS Official Method Ce 6-86 “Antioxidants, Liquid Chromatographic Method” was originally developed to confirm the correct antioxidant was added at the specified concentration to refined oils. Today, this method is increasingly utilized to validate that antioxidants are absent from oil products. False positive results can have a significant impact on the ability to sell products in specific markets and can impart additional business expenditures for conclusive secondary analyses. In the current work, quantification of *tert*-butylhydroquinone (TBHQ) in crude canola/rapeseed oil using liquid chromatography (LC) with ultraviolet (UV) detection was compromised by an interfering peak. Analyses using liquid chromatography-mass spectrometry (GC–MS) and high-resolution accurate mass LC–MS identified the interferent as 2,6-dimethoxy-4-vinylphenol (canolol), an endogenous compound present in crude canola/rapeseed oil. Resolution of canolol and TBHQ using LC-UV can be achieved via minor modification of the chromatographic conditions.

## Introduction

Vegetable oils with unsaturated fatty acids are subject to oxidation reactions upon storage with the degree of oxidizability directly related to the level of unsaturation [1]. This oxidation reaction results in oils becoming rancid and unpalatable. Antioxidants have been added to oils as one means of ensuring oxidative stability. Common antioxidants used in vegetable oils include *tert*-butylhydroquinone (TBHQ), 2- and 3-*tert*-butyl-4-hydroxyanisole (BHA), and 3,5-di-*tert*-butyl-4-hydroxytoluene (BHT). Recently, regulatory guidelines have been implemented in some countries banning the use of synthetic antioxidants due to reported adverse health effects on humans exposed to them [2, 3].

Canola oil is produced from the seed of any of several cultivars of rapeseed bred to be low in erucic acid [4]. Canola was originally a trademark, but is now a generic term for edible varieties of rapeseed oil in North America and Australia. In Canada, an official definition of canola is codified in Canadian law [4]. Canola/rapeseed oil made from low glucosinolate-low erucic acid rapeseed is produced for food consumption and biodiesel production. It is the third most produced oil in the world after palm and soybean oil.

Archer Daniels Midland Company (ADM) is a global supplier of canola oil and routinely tests its products to ensure compliance with applicable regulations. To prove the absence of TBHQ in canola oil, several customers have required the assay to be performed in accordance with AOCS Official Method Ce 6-86 “Antioxidants, Liquid Chromatographic Method” [5]. This method, however, was developed to confirm the correct antioxidant was added at the specified concentration to refined oils, not to validate the absence of antioxidants in oil products. Using this method, a third-party contract laboratory recently reported positive results for TBHQ in crude canola oil samples. Although these samples were known to be free of any synthetic antioxidants, similar results were obtained in our laboratory using liquid chromatography with ultraviolet detection (LC-UV). In order to evaluate the extent of this issue, crude canola samples were collected from five major growing areas in North America and analyzed using AOCS Official Method Ce 6-86. All of the samples yielded a peak that co-eluted with TBHQ. This work was conducted to identify the TBHQ-interfering peak in crude canola oil, and to develop a chromatographic resolution to separate these analytes.

## Materials and Methods

AOCS Official Method Ce 6-86 can be used to quantify antioxidants in oils including propyl gallate, 2,4,5-trihydroxybutyrophenone (THBP), TBHQ, nordihydroguaiaraetic acid (NDGA), BHA, 2,6-di-*tert*-butyl-4-hydroxymethylphenol (Ionox-100), and BHT. Briefly, 20 g of oil are diluted to 100 mL with hexane. A 25-mL aliquot of this solution is partitioned with three 50-mL aliquots of acetonitrile. The acetonitrile extracts are combined and concentrated to 3–4 mL using flash evaporation and then brought to a final volume of 10 mL with 2-propanol. Sample analysis is performed via reversed phase chromatography with mobile phase *A* = 5% acetic acid in water and mobile phase *B* = 5% acetic acid in acetonitrile. The mobile phase gradient is linear from 30% (*B*) to 100% (*B*) over 10 min followed by a 4-min hold at 100% (*B*). In our laboratory, a YMC-Pack ODS-AM, 150 × 3.0 mm column with a 3-µm particle size has been substituted for the older Lichrosorb 250 × 4.6 mm column with mobile phase flow rate reduced from 2.0 to 0.65 mL/min. In order to resolve TBHQ and canolol, the mobile phase gradient was modified to 30% (*B*) to 40% (*B*) from 0 to 5.5 min, 40% (*B*) to 100% (*B*) from 5.5 to 6 min, followed by a 1.5-min hold at 100% (*B*).

### High-Resolution Accurate Mass LC–MS (HRAM LC–MS)

The same column was used as noted above but the mobile phases were modified to *A* = 0.1% formic acid in water and *B* = acetonitrile in order to avoid ion suppression with the high acetic acid concentrations. A UV detector was set up in series with a QExactive Orbitrap mass spectrometer (Thermo Fisher Scientific, Waltham, MA, USA). An APCI source was used in positive ion mode. Mass spectrometer settings were: sheath gas flow rate = 60; auxiliary gas flow rate = 20; sweep gas flow rate = 5 (all arbitrary units); discharge current = 4.0 µA; capillary temperature = 350 °C; S-lens RF level = 50; and vaporizer temperature = 350 °C.

### Gas Chromatography–Mass Spectrometry

Crude canola oil extracts were analyzed using a GC-2010 with a GCMS-QP2020 (Shimadzu Scientific Instruments, Inc. Columbia, MD, USA). Injection was conducted in split mode (1 µL, 5:1 split ratio, inlet temperature 340 °C), and components were chromatographed through a 5% diphenyl-modified polydimethylsiloxane capillary column (Agilent DB-5MS; 30-m length, 0.25-mm inner diameter, 0.25-µm film thickness) with helium as the carrier gas maintained at a constant linear velocity of 31.8 cm/s. The oven temperature profile was 75–345 °C with a ramp of 15 °C/min followed by a 15-min hold at 345 °C. The ion source temperature was 200 °C, and the transfer line was maintained at 345 °C. Electron ionization spectra were collected from 15 to 250 *m/z* at an ionization voltage of 70 eV.

## Results and Discussion

**Fig. 1:**
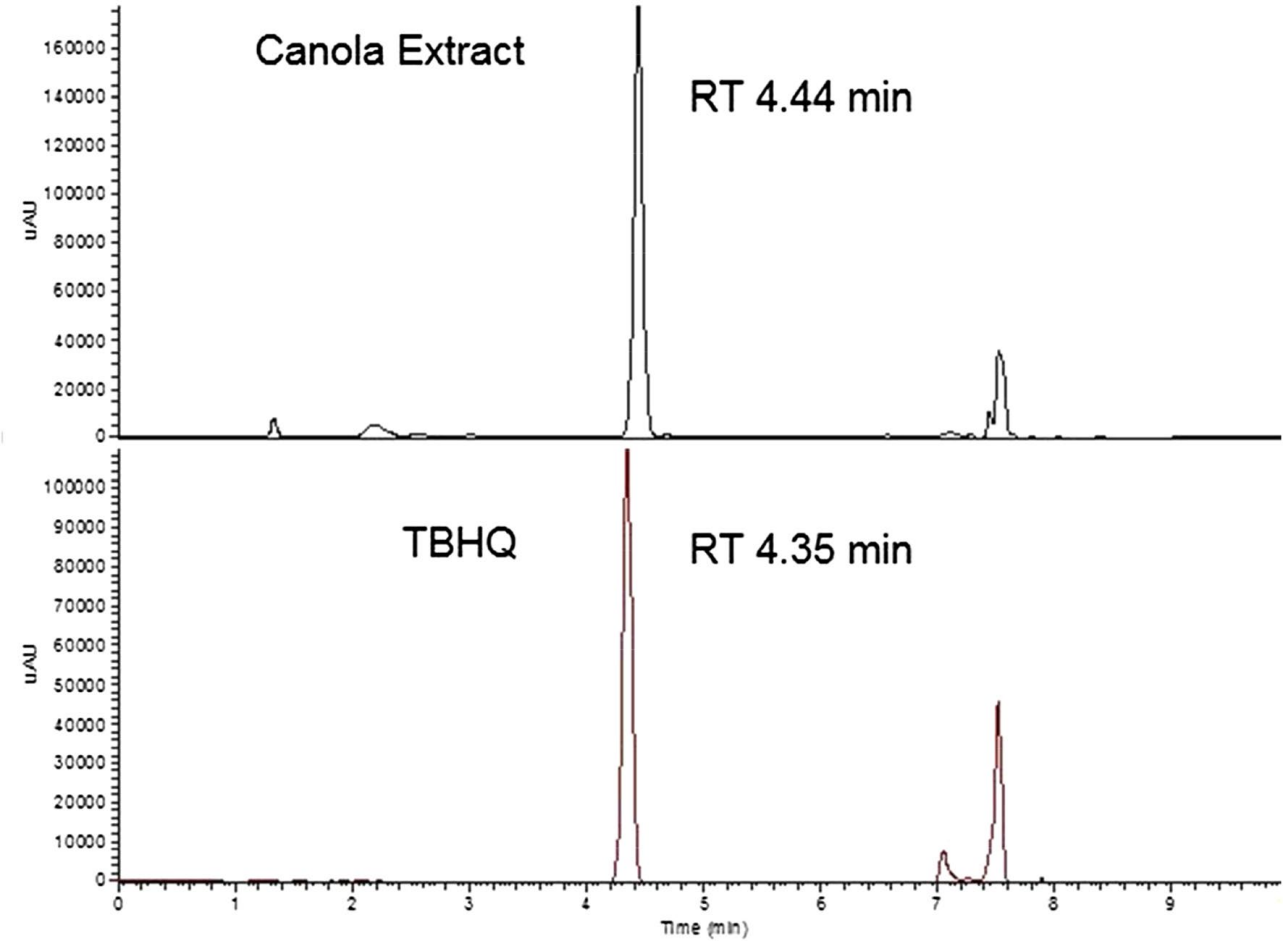
LC-UV trace at 280 nm of crude canola oil extract (*top*) and 200 ppm TBHQ calibration standard (*bottom*)

Initial analyses of crude canola oil using AOCS Method Ce 6-86 showed the presence of an apparent TBHQ peak at concentrations greater than 450 mg/kg despite the fact that no TBHQ had been added to this product (Fig. 1). These results were in agreement with a third-party contract laboratory using AOCS Official Method Ce 6-86 (data not shown).

**Fig. 2:**
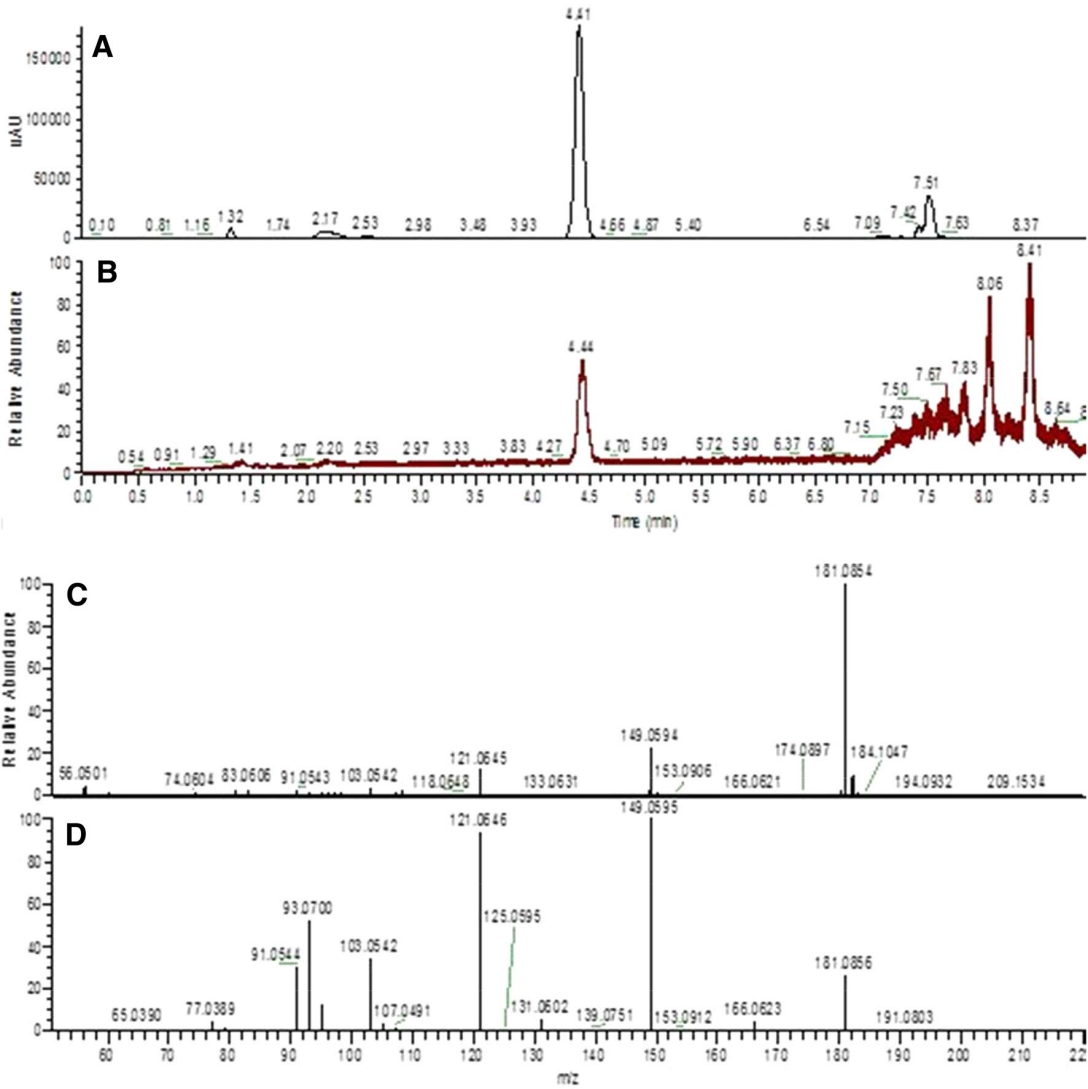
LC-UV trace at 280 nm of crude canola oil extract (**a**); LC–MS total ion chromatogram of canola oil extract (**b**); full scan, positive ion spectra of peak at 4.4 min (**c**); and MS/MS, positive ion spectra of peak at 4.4 min (**d**)

Samples were then assayed using HRAM LC-MS to confirm TBHQ levels after modification of the mobile phases. Retention time of the UV peak with absorption at 280 nm increased from 3.5 to 4.4 min with the weaker acid mobile phase (Fig. 2), but mass spectrometry showed this peak was not TBHQ. Full scan LC–MS analysis of this peak yielded a *m/z* of 181.0858 and best-fit formula of C_10_H_12_O_3_ (Fig. 2). The fragmentation pattern of this ion is shown in Fig. 2d, but a search of METLIN™ and mzCloud™ databases^1^yielded no conclusive identification of this compound.

**Fig. 3:**
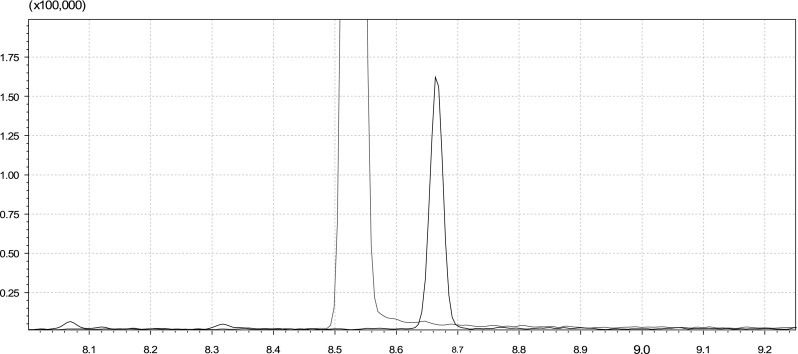
Expanded GC–MS total ion chromatogram overlay comparing an acetonitrile extract of crude canola oil (*black trace*) with a standard of TBHQ in acetonitrile (*red trace*)

Analysis of the crude canola oil sample using GC–MS produced a total ion chromatogram with an unknown peak eluting close to TBHQ (Fig. 3). This compound also had an apparent molecular formula of C_10_H_12_O_3_, and its fragmentation pattern was a close match to that of canolol, although some of the lower-intensity peaks detected in the unknown compound were missing in the library spectrum.

**Fig. 4:**
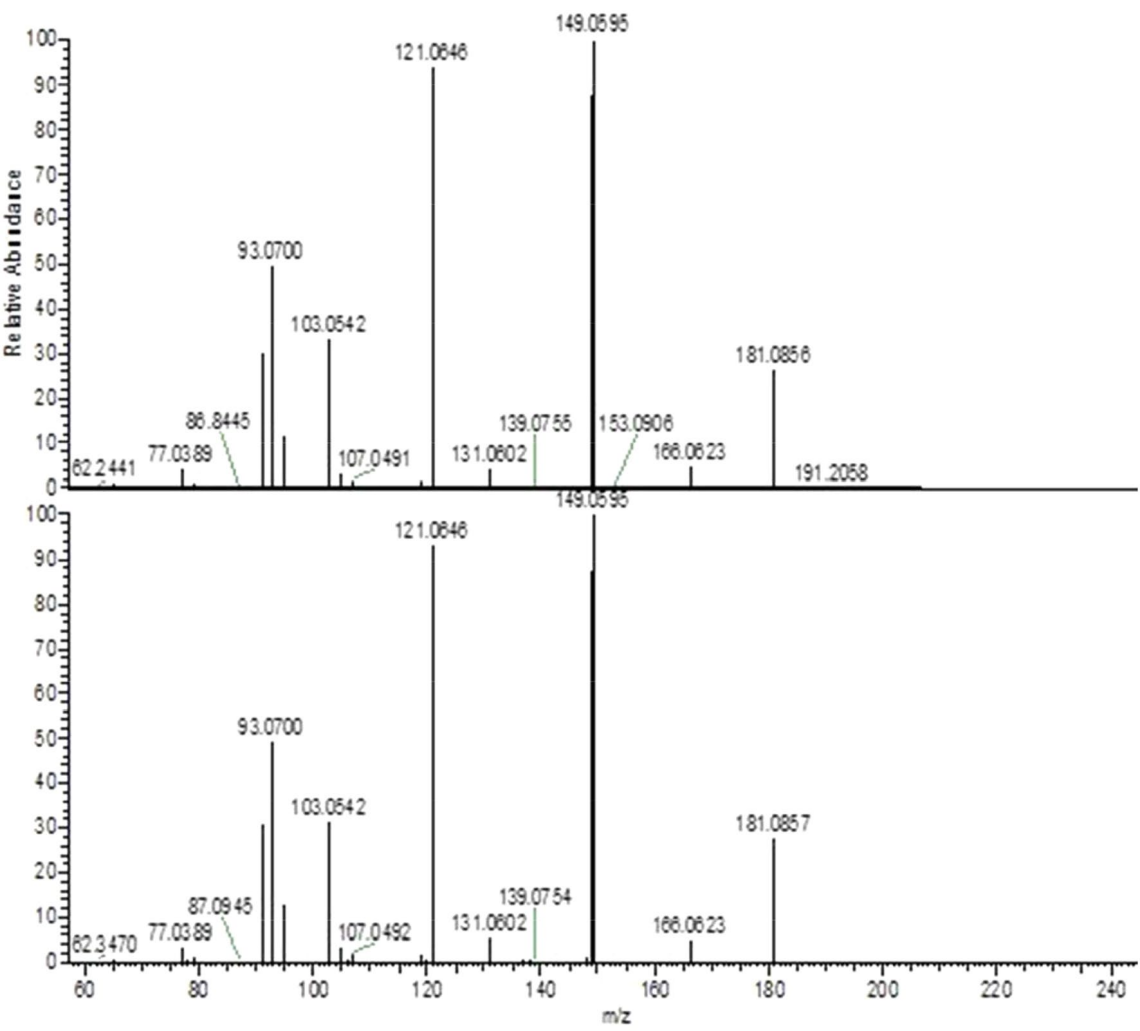
LC–MS/MS fragmentation of crude canola oil unknown (*top*) and canolol (*bottom*) using a normalized collision energy of 20

**Fig. 5:**
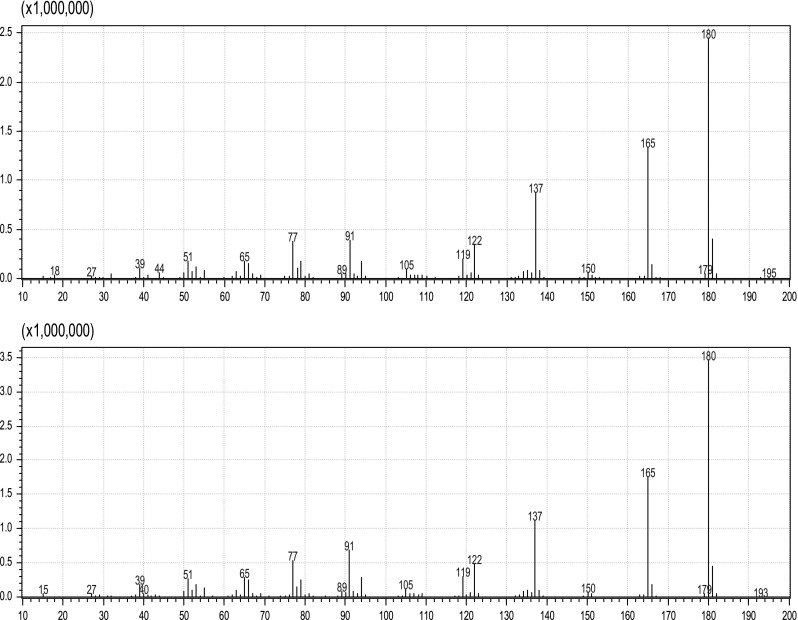
GC–MS/MS fragmentation of crude canola oil unknown (*top*) and canolol (*bottom*)

Based on this information, a canolol reference standard was purchased (Ryan Scientific, Inc. Mt. Pleasant, SC, USA) and assayed using the same LC–MS/MS and GC–MS/MS conditions used for the canola oil unknown. The fragmentation patterns of canolol and the unknown peak in canola oil yielded exact matches using both LC–MS/MS (Fig. 4) and GC–MS/MS (Fig. 5), confirming its identity. Canolol is an endogenous component of rapeseed oil [6], but its effect on antioxidant analyses in canola oil has not been documented.

## Conclusions

**Fig. 6:**
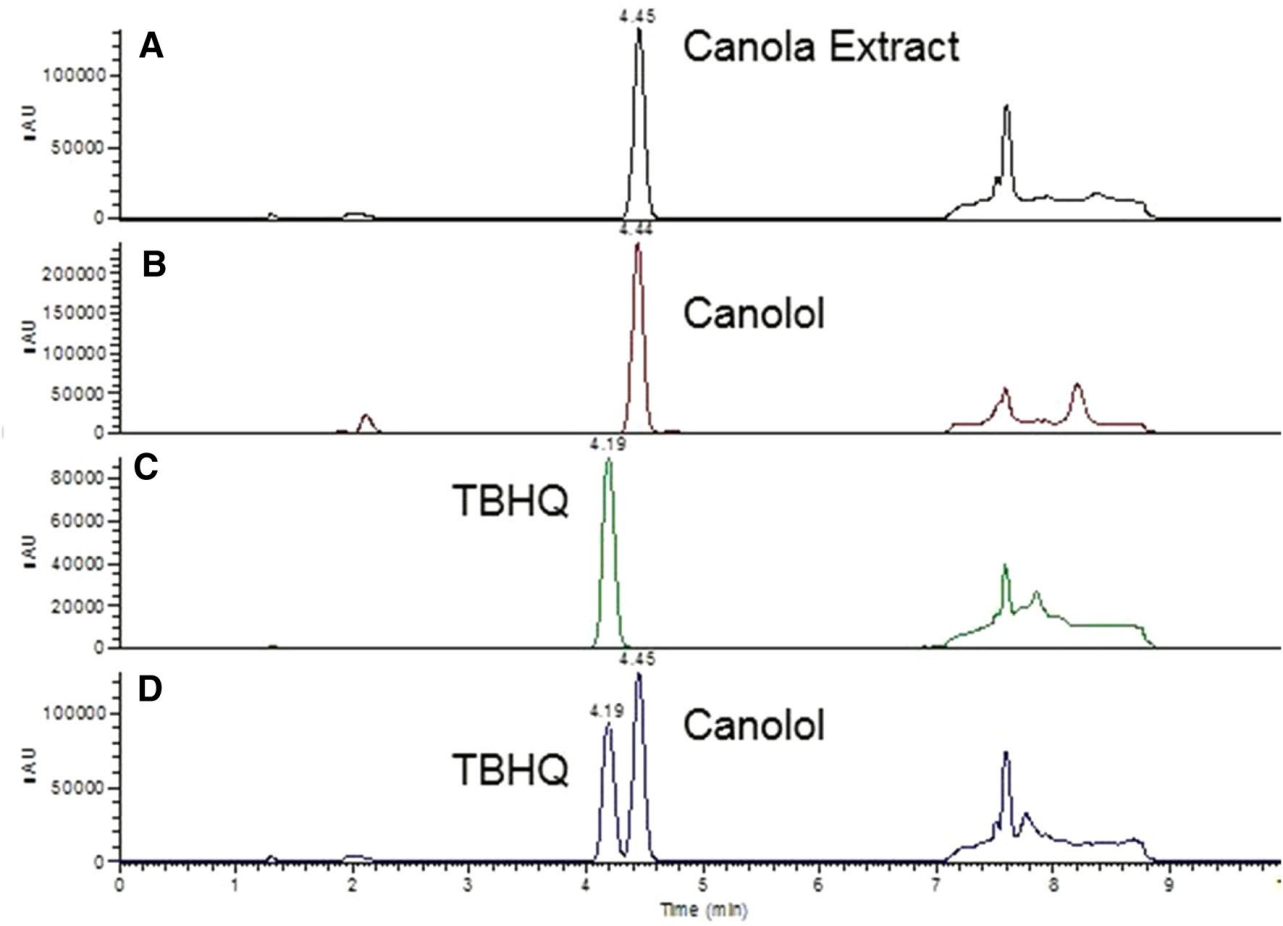
LC-UV (280 nm) of crude canola extract (**a**); canolol calibration standard (**b**); TBHQ calibration standard (**c**); and crude canola extract spiked with TBHQ (**d**) using a modified mobile phase gradient

This data confirmed that TBHQ quantification in crude canola oil is subject to interference from canolol when using AOCS Official Method Ce 6-86 as written. Modification of the gradient method to a shallower slope improves resolution of TBHQ and canolol. The gradient used to generate chromatograms in Fig. 6 was 30% (*B*) to 40% (*B*) from 0 to 5.5 min, 40% (*B*) to 100% (*B*) from 5.5 to 6 min, followed by a 1.5-min hold at 100% (*B*). This method provides adequate resolution of TBHQ and canolol using an overall analysis time similar to that described in AOCS Official Method Ce 6-86.

## Footnotes

1 METLIN is a registered trademark of the Scripps Research Institute, La Jolla, CA. mzCloud is a registered trademark of HighChem LLC, Slovakia.
